# The Protective Role of HLA-DRB1^∗^13 in Autoimmune Diseases

**DOI:** 10.1155/2015/948723

**Published:** 2015-10-29

**Authors:** Andreia Bettencourt, Cláudia Carvalho, Bárbara Leal, Sandra Brás, Dina Lopes, Ana Martins da Silva, Ernestina Santos, Tiago Torres, Isabel Almeida, Fátima Farinha, Paulo Barbosa, António Marinho, Manuela Selores, João Correia, Carlos Vasconcelos, Paulo P. Costa, Berta Martins da Silva

**Affiliations:** ^1^Immunogenetics Laboratory, Instituto de Ciências Biomédicas Abel Salazar-Universidade do Porto (ICBAS-UP), Rua de Jorge Viterbo Ferreira No. 228, 4050-313 Porto, Portugal; ^2^Unit for Multidisciplinary Research in Biomedicine (UMIB), Abel Salazar Institute of Biomedical Sciences (ICBAS), University of Porto (UP), Rua de Jorge Viterbo Ferreira No. 228, 4050-313 Porto, Portugal; ^3^Department of Neurology, Centro Hospitalar do Porto-Hospital de Santo António (CHP-HSA), Largo Prof. Abel Salazar, 4099-001 Porto, Portugal; ^4^Unidade de Imunologia Clínica (UIC), Centro Hospitalar do Porto-Hospital de Santo António (CHP-HSA), Largo Prof. Abel Salazar, 4099-001 Porto, Portugal; ^5^Department of Dermatology, Centro Hospitalar do Porto-Hospital de Santo António (CHP-HSA), Largo Prof. Abel Salazar, 4099-001 Porto, Portugal; ^6^Department of Internal Medicine, Centro Hospitalar do Porto-Hospital de Santo António (CHP-HSA), Largo Prof. Abel Salazar, 4099-001 Porto, Portugal; ^7^Instituto Nacional de Saúde Dr. Ricardo Jorge (INSA), Rua Alexandre Herculano No. 321, 4000-055 Porto, Portugal

## Abstract

Autoimmune diseases (AIDs) are characterized by a multifactorial aetiology and a complex genetic background, with the MHC region playing a major role. We genotyped for HLA-DRB1 locus 1228 patients with AIDs-213 with Systemic Lupus Erythematosus (SLE), 166 with Psoriasis or Psoriatic Arthritis (Ps + PsA), 153 with Rheumatoid Arthritis (RA), 67 with Systemic Sclerosis (SSc), 536 with Multiple Sclerosis (MS), and 93 with Myasthenia Gravis (MG) and 282 unrelated controls. We confirmed previously established associations of HLA-DRB1^∗^15 (OR = 2.17) and HLA-DRB1^∗^03 (OR = 1.81) alleles with MS, HLA-DRB1^∗^03 with SLE (OR = 2.49), HLA-DRB1^∗^01 (OR = 1.79) and HLA-DRB1^∗^04 (OR = 2.81) with RA, HLA-DRB1^∗^07 with Ps + PsA (OR = 1.79), HLA-DRB1^∗^01 (OR = 2.28) and HLA-DRB1^∗^08 (OR = 3.01) with SSc, and HLA-DRB1^∗^03 with MG (OR = 2.98). We further observed a consistent negative association of HLA-DRB1^∗^13 allele with SLE, Ps + PsA, RA, and SSc (18.3%, 19.3%, 16.3%, and 11.9%, resp., versus 29.8% in controls). HLA-DRB1^∗^13 frequency in the AIDs group was 20.0% (OR = 0.58). Although different alleles were associated with particular AIDs, the same allele, HLA-DRB1^∗^13, was underrepresented in all of the six diseases analysed. This observation suggests that this allele may confer protection for AIDs, particularly for systemic and rheumatic disease. The protective effect of HLA-DRB1^∗^13 could be explained by a more proficient antigen presentation by these molecules, favouring efficient clonal deletion during thymic selection.

## 1. Introduction

Autoimmune diseases (AIDs) are chronic disorders originated by the loss of immunological tolerance to self-antigens. This heterogeneous group of conditions present common genetic risk factors and share several pathophysiological mechanisms leading to overlapping clinical manifestations targeting specific organs or multiple organ systems [1]. There is evidence that they share similar immunogenetic mechanisms, even though they exhibit varying epidemiological features and clinical manifestations [2, 3]. Underlying these diverse clinical phenotypes is a deregulated immune system with an enriched ability to respond against self-tissues. The fact that AIDs share several clinical signs and symptoms (i.e., subphenotypes) and also share physiopathological mechanisms and genetic factors has been called autoimmune tautology and indicates that they may have a common origin [4].

The immune system is in charge of the defence against external pathogens. For this purpose, T and B lymphocytes are responsible for the immune response through regulated cell-cell interactions and secretion of cytokines, chemokines, and other inflammatory mediators. This defence against external pathogens must occur without causing unnecessary harm to self. To achieve this delicate balance, the majority of self-reactive T and B lymphocytes are destroyed in the thymus and bone marrow through negative selection [5]. Nevertheless, this process is far from perfect, and self-reactive lymphocytes escape into the periphery. Consequently, peripheral tolerance mechanisms are necessary to keep these self-reactive cells in check [6]. Activated self-reactive T and B cells promote autoimmunity when the effector and regulatory balance of the immune response is disturbed [7].

Major histocompatibility complex (MHC) molecules are widely distributed surface membrane glycoproteins that present antigenic peptides to T cell receptors (TCRs). Developing thymocytes encounter a highly heterogeneous repertoire of self (endogenous) peptide-MHC (pMHC) complexes on thymic epithelial cells, the main thymus antigen presenting cells. The affinity/avidity with which these thymocyte TCRs bind self pMHC determines if it is destined to perish or if it will survive [8]. In this way, a repertoire of peripheral T cells that is essentially self-tolerant is generated [6, 9, 10].

Several hypotheses have been put forward to explain how MHC polymorphisms influence autoimmunity risk or protection. They must do so, somehow, by shaping the central or peripheral T cell repertoires toward autoimmune resistance or proclivity [8]. A protective MHC profile could achieve this by the selection of a T cell repertoire with diminished pathogenicity [11]. On the other hand, protective MHC molecules may keep autoimmunity in check by favouring the negative selection of particular self-reactive T cells [12–14].

The functional basis of the association between specific HLA alleles and development of AIDs can be classically explained by two possible etiopathogenic models [15].

*The molecular mimicry hypothesis* proposes that certain HLA alleles are more efficient in presenting pathogen epitopes that share structural features with self-peptides to mature T cells. Once the response to the pathogen is initiated the self-antigen is also recognized and disease ensues.

*Central selection failure* proposes that certain HLA alleles are less efficient at presenting self-peptides to developing T cells in the thymus, so negative selection fails.

A different hypothesis proposes that different alleles can modulate the immunologic profile of an individual, through antigen-independent mechanisms, resulting in either promoting a higher autoimmune predisposition or, in opposition, a more efficient immune regulation. Given the consistent association of HLA-DRB1 alleles with different autoimmune diseases (Table 1), we explored the idea that the same HLA-DRB1 alleles could be influencing several different autoimmune diseases. To this end we compared the immunogenetic profile in different AIDs. This study includes four autoimmune systemic diseases, namely, Systemic Lupus Erythematosus (SLE), Rheumatoid Arthritis (RA), Psoriasis or Psoriatic Arthritis (Ps + PsA), and Systemic Sclerosis (SSc). Patients with Multiple Sclerosis (MS) and Myasthenia Gravis (MG) were also included.

## 2. Patients and Methods

### 2.1. Patients and Controls

A total of 1228 patients with AIDs, 213 patients with SLE and 153 patients with RA diagnosed according to the American College of Rheumatology (ACR) criteria, 166 patients with Ps + PsA, 67 with SSc, 536 with definitive diagnosis of MS according to the revised McDonald criteria, and 93 with MG, were recruited from the Neurology and Medicine Outpatient Clinic of Centro Hospitalar do Porto-Hospital de Santo António (CHP-HSA). The HLA-DRB1 frequencies of patients were compared with the ones of a control group consisting of 282 unrelated individuals without disease and from the same geographic origin (north of Portugal).

### 2.2. HLA-DRB1 Genotyping

Peripheral blood samples (10 mL) were collected in EDTA. Genomic DNA was obtained from proteinase-K–treated peripheral blood leukocytes by using a Salting-Out procedure [27]. Low-resolution genotyping for HLA–DRB1 locus (i.e., 2-digit HLA nomenclature) was performed using polymerase chain reaction and sequence-specific primers (PCR-SSP), based on methods previously described [28]. In order to produce PCR-SSP reactions able to detect and discriminate each of the known HLA-DRB1 genes, primers were designed using sequence alignments comprising all HLA-DRB1 variants and were validated by the Twelfth International Histocompatibility Workshop. PCR products were visualized under ultraviolet light after running in a 1.5% agarose gel containing ethidium bromide.

### 2.3. Statistical Analysis

To identify the HLA-DRB1 genes contributing to the six different AIDs, we applied stepwise logistic regression on an allelic level, using forward selection which involves starting with no variables in the model, testing the addition of each variable using a chosen model comparison criterion, adding the variable (if any) that improves the model the most, and repeating this process until none improves the model. It should be noted that odds ratios (ORs) obtained in a multivariable logistic regression analysis are adjusted for all the other genes included in the model and therefore differ from those obtained when a given gene is compared with all other genes. The data were analysed using IBM SPSS 20 statistical software.

## 3. Results

A total of 1228 cases and 282 controls were analysed and different types of association between alleles and AIDs were found (Table 2). These included three risk alleles for two or more AIDs, two protective alleles for two or more AIDs, and three risk alleles for a particular AID.

HLA-DRB1^*∗*^13 was a protective allele for four AIDs: SLE (18.3% versus 29.8%, *p* = 0.016, OR = 0.58, and 95% CI = 0.37–0.90), Ps + PsA (19.3% versus 29.8%, *p* = 0.050, OR = 0.621, and 95% CI = 0.39–1.00), RA (16.3% versus 29.8%, *p* = 0.044, OR = 0.58, and 95% CI = 0.34–0.98), and SSc (11.9% versus 29.8%, *p* = 0.035, OR = 0.42, and 95% CI = 0.19–0.94).

There was a specific risk allele associated with three AIDs. HLA-DRB1^*∗*^03 was found to be a risk factor for SLE (34.3% versus 15.6%, *p* = 4.2 × 10^−5^, OR = 2.49, and 95% CI = 1.61–3.86), MS (22.9% versus 15.6%, *p* = 0.003, OR = 1.81, and 95% CI = 1.23–2.67), and MG (35.5% versus 15.6%, *p* = 6.1 × 10^−5^, OR = 2.98, and 95% CI = 1.75–5.07). There were two risk alleles associated with two AIDs: HLA-DRB1^*∗*^08 was positively associated with MS (12.1% versus 8.5%, *p* = 0.033, OR = 1.73, and 95% CI = 1.05–2.87) and SSc (22.4% versus 8.5%, *p* = 0.004, OR = 3.01, and 95% CI = 1.43–6.31) and HLA-DRB1^*∗*^01 was found to be a risk factor for RA (32.7% versus 23.4%, *p* = 0.017, OR = 1.79, and 95% CI = 1.11–2.88) and SSc (41.8% versus 23.4%, *p* = 0.006, OR = 2.28, and 95% CI = 1.27–4.09).

HLA-DRB1^*∗*^09 was negatively associated with SLE (1.0% versus 5.0%, *p* = 0.027, OR = 0.18, and 95% CI = 0.04–0.83), MS (1.0% versus 5.0%, *p* = 0.004, OR = 0.22, and 95% CI = 0.08–0.63), and RA (0.0% versus 1.0%, *p* = 0.003, OR = 0.95, and 95% CI = 0.93–0.98).

Three risk disease-specific alleles were found: HLA-DRB1^*∗*^04 for RA (47.7% versus 24.5%, *p* = 6 × 10^−6^, OR = 2.81, and 95% CI = 1.79–4.39), HLA-DRB1^*∗*^07 for Ps + PsA (39.8% versus 25.5%, *p* = 0.006, OR = 1.79, and 95% CI = 1.18–2.72), and HLA-DRB1^*∗*^15 for MS (32.7% versus 19.9%, *p* = 2 × 10^−5^, OR = 2.17, and 95% CI = 1.53–3.10).

Considering AIDs as a group, HLA-DRB1^*∗*^03 frequency was significantly higher (23.9% versus 15.6%, *p* = 0.022, OR = 1.51, and 95% CI = 1.0–2.15) compared with controls; conversely HLA-DRB1^*∗*^13 (20.0% versus 29.8%, *p* = 0.004, OR = 0.58, and 95% CI = 0.43–0.79) and HLA-DRB1^*∗*^09 (1.4% versus 5.0%, *p* = 1 × 10^−4^, OR = 0.23, and 95% CI = 0.11–0.49) frequencies were significantly lower.

## 4. Discussion

Through a systematic review of published works, Cruz-Tapias and collaborators, in 2012, identified some common HLA class II alleles that contribute to susceptibility to AIDs in Latin Americans [3]. The present study is, to date and to the best of our knowledge, the only one that addresses the hypothesis that a HLA-DRB1 allele could influence different autoimmune diseases, using a new cohort, encompassing six different autoimmune diseases.

In this study we observed associations of different HLA-DRB1 alleles with several AIDs. We confirmed positive and negative associations in MS [24, 25], SLE [16–18], Ps + PsA [19, 20], RA [21], SSc [22, 23], and MG [26], previously reported in our or other populations.

When AIDs studied were considered as a group, HLA-DRB1^*∗*^03 allele was significantly overrepresented, as already described [29]. It has been shown that this allele has low affinity for CLIP (class II-associated invariant chain peptide) and may not require HLA-DM to ensure peptide presentation, preventing efficient peptide selection and allowing the binding of low stability peptides [30]. Concerning the observed negative association with HLA-DRB1^*∗*^09, we think that this is likely a spurious association, as this is a rare allele and the frequency found in controls is, for some reason, double the one reported for the Portuguese population [31].

Our observations suggest that the presence of HLA-DRB1^*∗*^13 allele may confer protection for AIDs. HLA-DRB1^*∗*^13 is a high frequency allele in the general population both in Portugal [31] and worldwide. Our results confirm that the lower frequency of HLA-DRB1^*∗*^13 in every individual AIDs group is not secondary to the deviations granted by the concurrent positive associations. When the data obtained from previous studies are taken into consideration, the HLA-DRB1^*∗*^13 allele seems to be a universal protective allele for RA. It was reported as protective against RA in Asian [32, 33], Turkish [34], and several European populations [35–37]. Recently this allele was also described to be protective in SLE in the Japanese population [18] and for ANCA-associated vasculitis in the Dutch population [38].

Subtle structural differences in the HLA molecule have functional implications at the protein level. Specific amino acid patterns at the peptide binding cleft are involved in disease susceptibility, such as the well-known shared epitope first described in the RA susceptibility alleles HLA-DRB1^*∗*^01 and HLA-DRB1^*∗*^04 [37, 39]. Similar to the shared epitope classification of susceptibility alleles, protective HLA-DRB1 alleles have been categorized according to several models. One of the most accepted classifications proposes that protection against RA is conferred by the DERAA sequence at positions 70–74 of the HLA-DRB1 allele [40]. Other models suggest that protection is conferred by an aspartic acid at position 70 (D70 allele) [41] or an isoleucine at position 67 (I67 allele) of the HLA-DRB1 molecule. Because it was unclear which HLA-DRB1 alleles were protective a meta-analysis was performed involving four European populations with >2,700 patients and >3,000 control subjects. The objective was to investigate exhaustively which HLA-DRB1 alleles were associated with protection against RA [36]. Interestingly, this study showed that the protective effect attributed to DERAA and D70 was no longer present after the exclusion of HLA-DRB1^*∗*^13. The authors concluded that this evidence indicates that HLA-DRB1^*∗*^13 rather than DERAA, D70, or I67 is associated with protection [36]. In a recent study van Heemst and collaborators identify citrullinated vinculin, present in the joints of ACPA^+^ RA patients, as an autoantigen targeted by ACPA and CD4^+^ T cells. These T cells recognize an epitope with the core sequence DERAA, which is also found in many microbes and in protective HLA-DRB1^*∗*^13 molecules, presented by predisposing HLA-DQ molecules. Intriguingly, DERAA-directed T cells were not detected in HLA-DRB1^*∗*^13^+^ donors, indicating that the DERAA epitope from HLA-DRB1^*∗*^13 could mediate thymic tolerance in these donors and explain the protective effects associated with HLA-DRB1^*∗*^13. They suggest that subjects born with HLA-DRB1^*∗*^13 will present the HLA-DRB1^*∗*^13-derived DERAA-peptide in the thymus, leading to tolerization of the DERAA-reactive T cell response [42]. The negative association we describe here supports the idea that the HLA-DRB1^*∗*^13 allele, possibly by its specific structural features, may as well confer resistance to several other AIDs. The protective effect of HLA-DRB1^*∗*^13 could be explained by a more proficient antigen presentation by these molecules [43, 44], favouring an efficient thymic selection. As a result, negative selection and development of DR-driven autoreactive regulatory T cells are promoted [8].

A different model would relate HLA molecules with the presence of specific endophenotypes indirectly associated with autoimmunity. Other studies of our group suggest that the HLA genotype may primarily influence the general activation state of CD4 T cells [45]. The protective effect of HLA-DRB1^*∗*^13 could also be explained by this effect. Curiously, several reports have suggested an association of HLA-DRB1^*∗*^13 and/or HLA-DQB1^*∗*^06 with slow disease progression in human immunodeficiency virus (HIV) infected individuals, meaning that among HIV controllers there is an association between the presence of certain class II HLA alleles and strong CD4 T cell responses [46, 47].

Although different alleles are associated with particular AIDs, the same allele, HLA-DRB1^*∗*^13, was underrepresented in all six diseases. This difference is statistically significant for the four rheumatic diseases studied. This observation suggests that this allele confers protection to AIDs in general and particularly to rheumatic diseases.

## Figures and Tables

**Table 1:** HLA-DRB1 alleles associated with SLE, Ps + PsA, RA, SSc, MS, and MG.

Autoimmune disease	HLA-DRB1 associated allele		References
	Susceptibility	Protection	
Systemic Lupus Erythematosus (SLE)	HLA-DRB1^*∗*^03 HLA-DRB1^*∗*^08 HLA-DRB1^*∗*^15	HLA-DRB1^*∗*^09 HLA-DRB1^*∗*^13	[16–18]

Psoriasis or Psoriatic Arthritis (Ps + PsA)	HLA-DRB1^*∗*^07	—	[19, 20]

Rheumatoid Arthritis (RA)	HLA-DRB1^*∗*^01 HLA-DRB1^*∗*^04 HLA-DRB1^*∗*^10	HLA-DRB1^*∗*^13	[21]

Systemic Sclerosis (SSc)	HLA-DRB1^*∗*^01 HLA-DRB1^*∗*^08 HLA-DRB1^*∗*^11	HLA-DRB1^*∗*^07 HLA-DRB1^*∗*^15	[22, 23]

Multiple Sclerosis (MS)	HLA-DRB1^*∗*^03 HLA-DRB1^*∗*^08 HLA-DRB1^*∗*^15	HLA-DRB1^*∗*^10 HLA-DRB1^*∗*^14	[24, 25]

Myasthenia Gravis (MG)	HLA-DRB1^*∗*^03	—	[26]

**Table 2:** Associations between HLA class II and six AIDs: SLE, Ps + PsA, RA, SSc, MS, and MG.

	Controls (*n* = 282)	SLE (*n* = 213)	Ps + PsA (*n* = 166)	RA (*n* = 153)	SSc (*n* = 67)	MS (*n* = 536)	MG (*n* = 93)	Total (*n* = 1228)
HLA-DRB1^*∗*^01	**66 (23.4%)**	40 (18.8%)	39 (23.5%)	**50 (32.7%)** **OR = 1.79 ** **p = 0.017**	**28 (41.8%)** **OR = 2.28 ** **p = 0.006**	100 (18.7%)	23 (24.7%)	280 (22.8%)

HLA-DRB1^*∗*^03	**44 (15.6%)**	**73 (34.3%)** **OR = 2.49 ** **p = 4.2 × 10** ^−**5**^	25 (15.1%)	28 (18.3%)	11 (16.4%)	**123 (22.9%)** **OR = 1.81** **p = 0.003**	**33 (35.5%)** **OR = 2.98** **p = 6.1 × 10** ^−**5**^	**293 (23.9%)** **OR = 1.51 ** **p = 0.022**

HLA-DRB1^*∗*^04	**69 (24.5%)**	42 (19.7%)	46 (27.7%)	**73 (47.7%) ** **OR = 2.81** **p = 6 × 10** ^−**6**^	13 (19.4%)	128 (23.9%)	23 (24.7%)	325 (26.5%)

HLA-DRB1^*∗*^07	**72 (25.5%)**	55 (25.8%)	**66 (39.8%)** **OR = 1.79 ** **p = 0.006**	38 (24.8%)	14 (20.9%)	147 (27.4%)	23 (24.7%)	343 (27.9%)

HLA-DRB1^*∗*^08	**24 (8.5%)**	21 (10.0%)	10 (6.0%)	**3 (2.0%) ** **OR = 0.24** **p = 0.026**	**15 (22.4%)** **OR = 3.01 ** **p = 0.004**	**65 (12.1%)** **OR = 1.73 ** **p = 0.033**	7 (7.5%)	121 (9.9%)

HLA-DRB1^*∗*^09	**14 (5.0%)**	**2 (1.0%)** **OR = 0.18 ** **p = 0.027**	5 (3.0%)	**0 (0.0%)** ^*∗*^ **OR = 0.95** **p = 0.003**	3 (4.5%)	**5 (1.0%)** **OR = 0.22 ** **p = 0.004**	2 (2.2%)	**17 (1.4%)** **OR = 0.23 ** **p = 1 × 10** ^−**4**^

HLA-DRB1^*∗*^13	**84 (29.8%)**	**39 (18.3%)** **OR = 0.58 ** **p = 0.016**	**32 (19.3%)** **OR = 0.62 ** **p = 0.050**	**25 (16.3%)** **OR = 0.58** **p = 0.044**	**8 (11.9%)** **OR = 0.42 ** **p = 0.035**	124 (23.1%)	17 (18.3%)	**245 (20.0%)** **OR = 0.58 ** **p = 0.004**

HLA-DRB1^*∗*^15	**56 (19.9%)**	55 (25.8%)	22 (13.3%)	17 (11.1%)	12 (17.9%)	**175 (32.7%)** **OR = 2.17 ** **p = 2 × 10** ^−**5**^	15 (16.1%)	296 (24.1%)

AIDs: autoimmune diseases; SLE: Systemic Lupus Erythematosus; Ps + PsA: Psoriasis or Psoriatic Arthritis; RA: Rheumatoid Arthritis; SSc: Systemic Sclerosis; MS: Multiple Sclerosis; MG: Myasthenia Gravis. ^*∗*^Fisher's exact test was used to calculate this value.
